# An Intravascular Magnetic Catheter Enables the Retrieval of Nanoagents from the Bloodstream

**DOI:** 10.1002/advs.201800807

**Published:** 2018-08-01

**Authors:** Veronica Iacovacci, Leonardo Ricotti, Edoardo Sinibaldi, Giovanni Signore, Fabio Vistoli, Arianna Menciassi

**Affiliations:** ^1^ The BioRobotics Institute Scuola Superiore Sant'Anna Piazza Martiri della Libertà, 33 56127 Pisa PI Italy; ^2^ Center for Micro‐BioRobotics @SSSA Istituto Italiano di Tecnologia Viale Rinaldo Piaggio 34 56025 Pontedera Italy; ^3^ Center of Nanotechnology Innovation@NEST Istituto Italiano di Tecnologia 56127 Pisa Italy; ^4^ NEST Scuola Normale Superiore and Istituto Nanoscienze‐CNR 56127 Pisa Italy; ^5^ Division of General and Transplant Surgery Azienda Ospedaliera Universitaria PisanaUniversity of Pisa Via Paradisa 2 56124 Pisa Italy

**Keywords:** intravascular devices, magnetic nanoparticles, magnetic retrieval, targeted therapy

## Abstract

The clinical adoption of nanoscale agents for targeted therapy is still hampered by the quest for a balance between therapy efficacy and side effects on healthy tissues, due to nanoparticle biodistribution and undesired drug accumulation issues. Here, an intravascular catheter able to efficiently retrieve from the bloodstream magnetic nanocarriers not contributing to therapy, thus minimizing their uncontrollable dispersion and consequently attenuating possible side effects, is proposed. The device consists of a miniature module, based on 27 permanent magnets arranged in two coaxial series, integrated into a clinically used 12 French catheter. This device can capture ≈94% and 78% of the unused agents when using as carriers 500 and 250 nm nominal diameter superparamagnetic iron oxide nanoparticles, respectively. This approach paves the way to the exploitation of new “high‐risk/high‐gain” drug formulations and supports the development of novel therapeutic strategies based on magnetic hyperthermia or magnetic microrobots.

Cancer is nowadays one of the main causes of mortality worldwide. Traditional cancer treatment approaches are based on systemic chemotherapy combined, whenever possible and effective, with surgical resection. This paradigm often implies only a partial tumor eradication, while side effects are tremendous, with a consequent dramatic reduction of the patient's quality of life.^1^, ^2^ Targeted therapy has the potential to revolutionize cancer treatment by enabling to penetrate into the hard‐to‐reach areas of the human body, selectively performing the therapy in a controllable fashion and thus increasing the therapeutic index (TI, defined as the ratio between the drug dose that produces toxicity in 50% of the population (TD50) and the minimum drug dose that is effective for the desired therapy for 50% of the population (ED50)).^3^

The role of nanomedicine in the targeting process has been relevant.^4^, ^5^, ^6^ Indeed, in the last two decades, a broad range of nanomaterials has been developed for targeted therapies.

The dream of all nanomedicine approaches is to develop the so‐called Ehrlich's “Magic Bullet,”^7^, ^8^ i.e., a drug that selectively attaches to diseased cells but nontoxic to healthy ones. Many efforts have been devoted toward the development of nanovectors able to efficiently disperse therapeutic molecules in their structures, to guarantee a favorable blood half‐life, and to minimize the immune system response.^9^ These nanovectors are supposed to actively recognize and bind to cancer cells or angiogenic endothelium surrounding the tumor, by exploiting specific affinity ligands.^10^ Some efforts have been also devoted to make therapeutic nanovectors responsive to endogenous (such as changes in pH, temperature, redox conditions, or enzymes' activity) or exogenous (such as magnetic fields, ultrasound, and various types of irradiation) stimuli,^11^ in order to deliver therapy on demand^12^ and furtherly increase the TI. Magnetic nanoparticles play a major role in nanomedicine for being intrinsically theranostic: they can act as contrast agents in magnetic resonance imaging, but they can be also used for active tumor targeting (by exploiting an external magnetic field source enabling accumulation at the site of interest) and active therapy triggering both to enable drug delivery and magnetic hyperthermia.^13^, ^14^, ^15^

However, magnetic nanoparticles' accurate tracking poses high challenges and has been concretely achieved mainly in lab setups, so far.^16^ Moreover, the associated targeting accuracy strongly depends on the envisaged working environment within the body. Particles released in realistic blood streams (as opposed to model flows in capillaries) are extremely hard to be accumulated at a target site by using magnets external to the body and relatively far from the vessel (e.g., 5–10 cm).^17^ As a matter of fact, the long‐term fate of magnetic particles injected in the bloodstream is hard to be controlled, and the associated risks of toxicity^18^, ^19^ limit their effective translation to the clinical practice.

While devising a novel targeted therapy approach, a proper balance between off‐ and on‐target drug accumulation^20^ should be pursued. Scarce accumulation at the tumor site, on average around 0.7% ,^21^ associated with the typical difficulties in overcoming physical biological barriers,^22^ results into ineffective therapy or into the need to inject high doses of nanocompounds to achieve the desired effects. On the other hand, biodistribution issues and potential toxicity of particles and drugs to healthy tissues must be taken into account. In fact, there is the risk of nullifying the advantages of targeted therapy, while restoring the same side effects of systemic drug administration. In this framework, the development of an efficient therapeutic paradigm appears as a never ending struggle between the need to concentrate toxic doses of therapeutics in the site of interest and the need to avoid undesired side effects on healthy tissues.

In order to avoid toxicity‐related issues and to regulate the long‐term fate of unaccumulated and unused nanovectors, two main strategies could be pursued: the employment of biodegradable and bioadsorbable nanocarriers^23^, ^24^, ^25^, ^26^, ^27^ or the implementation of retrieval strategies aimed at removing the unused agents. The main limitation of the first strategy lies in the fate of the loaded drug after carrier degradation: the unbound drug can be released in correspondence to the healthy tissues with the risk of running again into side effects related issues.

On the other hand, the development of an efficient retrieval strategy would enable to overcome such limitations. A proper retrieval strategy should be independent on the specific exploited compound and should be featured by high efficiency to enable to get out from the aforementioned struggle.

In this paper, we propose a high‐efficiency retrieval strategy to remove from the bloodstream therapeutics provided with magnetic properties (or bound to a magnetic nanoparticle) that were not accumulated at the target site. It is based on an intravascular magnetic catheter devised to access those organs that feature a terminal circulation. This intravascular tool represents an enabling technology to minimize the uncontrollable dispersion of potentially toxic agents not contributing to therapy, thus considerably attenuating side effects.

We designed, developed, and tested an intravascular magnetic tool able to access organs featured by a terminal circulation such as liver, pancreas, lung, and kidney. For these organs, it is possible to clearly identify a main arterial inlet and a main venous outlet. This peculiar anatomy can be thus exploited to achieve a double access to the target district. On one hand, the arterial inlet enables the infusion of the therapeutic agents directly into the target organ, thus to favor accumulation in the region of interest (e.g., a liver carcinoma) and to limit its spreading in other districts. On the other hand, the venous access enables the capture of the therapeutic vectors that do not accumulate at the tumor site and that exit the target organ (**Figure** **1**a, Movie S1, Supporting Information). The liver was identified as a suitable and relevant case study due to its favorable vascular anatomy, to the high incidence of liver carcinoma, and to the limitations of traditional resection and pharmacological strategies.^28^, ^29^ In this case study, the hepatic artery can be targeted through a traditional 3–5 French (F) catheter for therapeutics injection, whereas access to the suprahepatic vein can be pursued by keeping retrieval tool dimensions compliant with a 12 F catheter. More precisely, in the case of hepatic tumor targeting, the arterial injection catheter is inserted in the femoral artery to reach backward the right or left hepatic artery or a segmental branch of one of them; whereas the magnetic catheter is inserted in the jugular vein to get to the correspondent right, median, or left suprahepatic vein according to the tumor venous drainage of each single patient. In this scenario, the proposed therapeutic procedure can be conceived as organized in the following sequence of actions:

1. The injection catheter is inserted across a main arterial access and brought selectively to the main artery feeding the target tumor in the organ.
2. The magnetic retrieval catheter is inserted across a main venous access and put in place in the main output vein collecting most of the blood exiting the lobe where the tumor grew in the target organ.
3. The magnetic nanoparticles bolus is injected through the arterial catheter to perform therapy (either accumulated by external magnetic fields favoring targeting, or based on specific ligand–receptor affinity).
4. The retrieval venous catheter catches the magnetic nanoparticles not accumulated into the tumor and thus not contributing to the therapy, removing them from the bloodstream.

**Figure 1:**
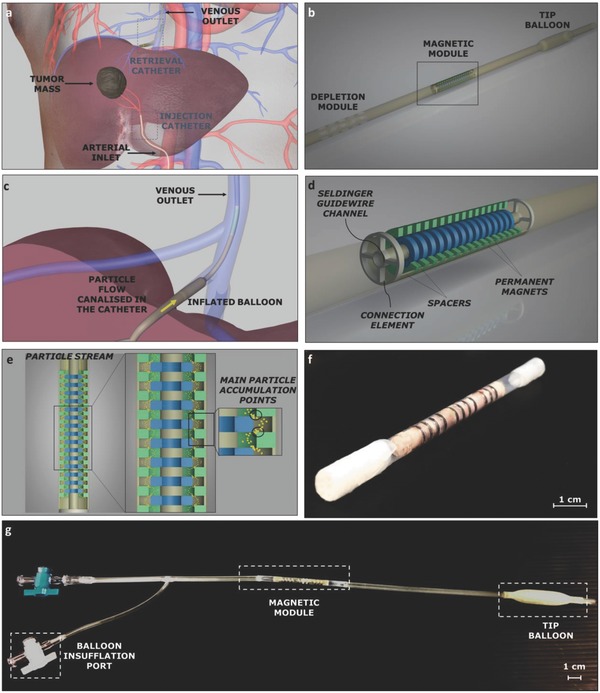
Intravascular retrieval catheter concept, structure and prototype. a) Depiction of a liver with a tumor mass and of the double access through the injection and retrieval catheters. b) Intravascular device schematization with constitutive blocks. c) Proposed catheter in the target vessel with the tip balloon in the inflated configuration and particle flow canalization (zoomed view of panel a). d) Extended view of the magnetic module. e) Magnetic particle trajectories (yellow dotted line) within the magnetic module. f) Magnetic module prototype. g) Retrieval catheter prototype.

The proposed intravascular device included three main units (Figure 1b): i) an inflatable balloon placed at the catheter tip, enabling anchoring at the target vessel and blood flow canalization inside the intravascular device (Figure 1c, Movies S2,S3, Supporting Information); ii) a magnetic module able to capture magnetic nanoagents; iii) a depletion segment allowing canalized blood reinsertion in the systemic circulation, after the “filtration” carried out by magnetic module. The catheter was provided with a central channel (diameter: 1 mm) along its overall length, thus enabling the sliding of a Seldinger guidewire (typically 0.89 mm in diameter for a 12 F catheter) and allowing catheter positioning in the target blood vessel.

The magnetic module represents the key unit of the device: it was designed in order to maximize capture efficiency while respecting the severe size constraints imposed by the working district. It is based on two coaxial series of miniature permanent magnets (we excluded the possibility to use electromagnets, to avoid heat generation and powering issues). Each series included a number of ring‐shaped permanent magnets, 15 in the central series and 12 in the external one, properly spaced by means of nonmagnetic elements, defined as spacers (Figure 1d). In the external series, magnets were featured by an outer diameter (o.d.) of 3.6 mm, an inner diameter (i.d.) of 2.4 mm, and a height (*h*) of 1 mm, whereas the central magnets showed an o.d. of 2.2 mm, an i.d. of 1 mm, and an *h* of 1 mm. The spacer wall thickness was considerably smaller than the permanent magnets one (200 vs 600 µm) both in the internal and the external series. Each series was preassembled by stacking consecutive magnets and spacers. Their final assembly was then achieved by a simple relative sliding, and secured through connection elements (Figure 1d). The blood flow was thus forced to follow the gap defined by the sagittal profile of the magnets: capture efficiency along this gap was maximized by leveraging magnetic gradient effects (Figure 1e, Movie S4, Supporting Information).

The magnetic module was fabricated by assembling customized permanent magnets (NdFeB N52 grade, axial magnetization, A.C. Magnets 98, S.L., Barcelona, Spain) and 3D‐printed spacers and connection elements. The whole structure was then integrated into a thermoplastic sheath. To this purpose, a bistable heat‐shrinkable fluorinated ethylene propylene extruded tube (ZEUS, USA), featured by a transition temperature around 215 °C, was used. This material is typically used to fabricate vascular catheters. The thermally triggered bistability of this element allowed a simple encapsulation of the magnetic module, with neither requiring complex assembly procedures nor implying a significant alteration of the overall diameter (the tube wall thickness was 100 µm; Figure 1f). A complete prototype was fabricated by properly modifying an aortic balloon catheter employed in aortic aneurysm surgery (Figure 1g), so as to integrate the magnetic retrieval module.

The magnetic module design was pursued to maximize nanoparticles capture efficiency. Finite element method (FEM) numerical simulations were performed to this aim, by means of a commercial tool (COMSOL Multiphysics). Device outcomes in terms of capture efficiency produced by articulated magnet arrangements in the catheter were investigated, by varying the design parameters (magnets number, shape, size, and arrangement). Simulations included both the magnetic field generated by the module and the blood fluid dynamics. In addition, particles were tracked through a Lagrangian method (**Figure** **2**a–e). By analyzing simulation results, it is possible to draw some considerations on system physics. Simulated blood speed was significantly affected by magnets (Figure 2c): the maximum speed value corresponded to the minimum gap section (by mass continuity). Magnetic field streamlines distribution resulted spatially complex, due to the superposition of adjacent magnets effects (Figure 2d). Particle capture was enhanced by gradient effects in proximity of the magnet edges (Figure 2e), whereas it was weakly dependent on magnets height. FEM simulations also revealed that permanent magnets placed at the center of the magnetic module played a more important role (contributing more to magnetic nanoparticles capture) with respect to magnets placed on the external catheter wall (Figure 2f and Figure S1, Supporting Information). The combination of two coaxial series of magnets resulted extremely advantageous: capture efficiency increased from 48% to 92% while varying the number of the embedded permanent magnets from 2 to 27, but with a slower trend above 12–14 magnets (Figure 2g and Figure S2, Supporting Information). Simulation results also shed light on the role played by magnet arrangement and on the distance between magnetic elements. By considering a fixed overall magnet number equal to 27, different grouping alternatives were evaluated, to clarify the role played by periodicity and clustering for magnetic nanoparticles capture (Figure 2h). Optimal capture efficiency was accomplished with three magnet groups. This arrangement was implemented in the intravascular device prototype.

**Figure 2:**
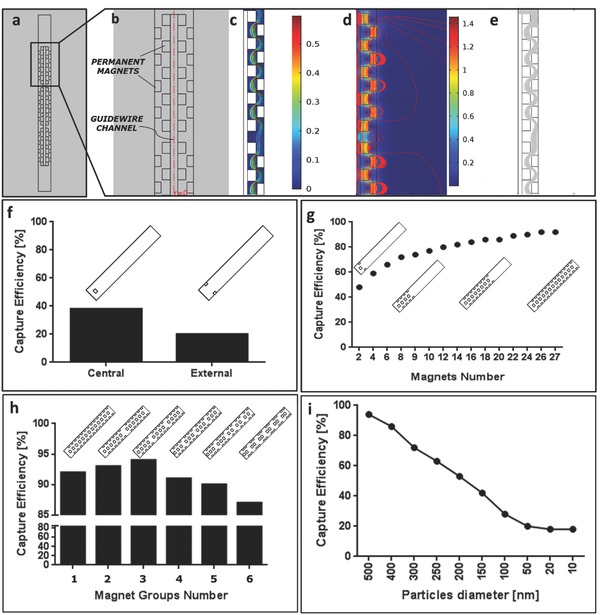
Multiphysics simulation results. a) Magnetic module 2D representation in COMSOL Multiphysics environment; b) zoomed view of a portion of the magnetic module in which the internal and the external series of permanent magnets are reported; c–e) Fluidic velocity, magnetic field, and particle trajectories in the proposed magnetic module; f) FEM simulations results in terms of capture efficiency when considering a single magnet, placed either centrally or externally, and 500 nm nominal diameter nanoparticles; magnetic capture efficiency when varying magnet number g), grouping h) or caught nanoparticle diameter i). The selected configuration is the number 3 in h).

In the optimal configuration, enabling 94% capture efficiency, the magnetic module included 27 ring‐shaped magnets organized in three subgroups and two coaxial series, for an overall module length of 33.7 mm. Retrieval efficiency was evaluated through FEM simulations carried out by considering the above‐mentioned catheter optimal configuration, while varying magnetic nanoparticle diameter from 500 down to 10 nm. Retrieval efficiency decreased almost linearly in the range 500–100 nm and reached a plateau around 20% capture efficiency for particle diameters below 50 nm (Figure 2i). It is worth mentioning that 500 nm is the maximum rigid carrier dimension enabling particle passage across the tiniest capillaries, while avoiding obstructions.^30^

FEM predictions were validated through in vitro magnetic retrieval experiments. To this purpose, a fluidic circuit connected to a micropump (M100 series, TCS) and a blood resembling solution (42% v/v glycerol in water^31^) were used. Retrieval tests were performed by injecting a 100 µL magnetic nanoparticles bolus before the magnetic module and then by collecting 30 mL of the fluid exiting from it. The nanoparticle injection point was selected far enough from the magnetic module thus to enable a proper mixing between the blood‐mimicking fluid and the magnetic nanoparticle solution, guaranteeing at the same time a proper distribution of the particles in the fluid profile. The micropump imposed the desired fluid velocity (7 cm s^−1^), corresponding to the simulated one and to the average physiological blood velocity in the suprahepatic vein. Finally, the embedding of a controlled valve inside the fluidic circuit (**Figure** **3**a) enabled to switch the circuit from a closed configuration to an open one, to allow sample collection.

**Figure 3:**
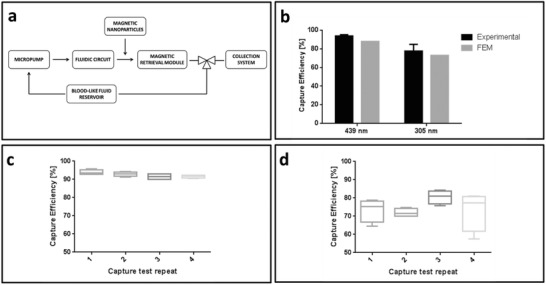
In vitro validation setup and results. a) In vitro validation fluidic circuit schematization. b) Comparison among theoretical (calculated through FEM) and experimental capture efficiency when considering 439 (nominal 500) and 305 (nominal 250) nm magnetic nanoparticles: experiments versus numerical simulations. Retrieval efficiency when performing multiple consecutive tests on the same prototype. Reported results refer to 500 nm c) and 250 nm d) nominal diameter magnetic nanoparticles.

Inductively coupled plasma mass spectrometry (ICP‐MS) allowed to quantitatively analyze the nanoparticle content in the collected samples, by correlating the Fe content with a standard curve. Retrieval efficiency was evaluated for 500 (09‐01‐502, Micromod, Germany) and 250 nm (09‐01‐252, Micromod, Germany) nominal diameter magnetic nanoparticles. Dynamic light scattering enabled to evaluate the effective particle diameter that resulted equal to 439 and 305 nm, respectively (Figure S3, Supporting Information).

Four module prototypes (featuring the optimal magnets configuration) were tested for each nanoparticle size. Results showed an average capture efficiency of 93.6% for 439 nm particles and 77.6% for 305 nm particles, in good agreement with FEM predictions (Figure 3b).

The overall system performance was also evaluated in presence of multiple nanoparticle doses. This test aimed at assessing if the magnetic catheter may allow multiple retrieval procedures by keeping its effectiveness. To investigate if saturation phenomena occurred, four consecutive tests were performed on each prototype, considering both 439 and 305 nm diameter particles. ICP‐MS analyses revealed that capture efficiency showed a slightly nonlinear decreasing trend, when increasing test repetitions. Nevertheless capture efficiency never fell below 89% and 65% for 439 and 305 nm magnetic particles, respectively, thus revealing a reliable retrieval efficiency of the proposed device, even when dealing with high doses and multiple usages (Figure 3c,d).

Further in vitro validation was performed to preliminarily assess the retrieval tool effects on blood rheology. Fresh whole blood samples from pig anticoagulated with EDTA were pumped at constant speed (7 cm s^−1^) in plain commercial catheters (control samples – C) and in the retrieval one (single passage samples – SP). A dedicated bench‐testing setup was employed to this aim; a controllable linear syringe pump was preferred to an electromechanical one to avoid hemolysis and hemorheological alterations due to the pump. Blood count analysis revealed that neither the red blood cells count (C: 7.42 ± 0.02 million per µL; SP: 7.23 ± 0.21 million per µL) nor the hematocrit (C: 43.07 ± 0.06%; SP: 42,6 ± 0.62%) were significantly altered by the retrieval tool, thus foreseeing negligible hemolysis. Similarly, no signs of platelet degradation were observed (C: 373.33 ± 16.07 thousand per µL; SP: 372 ± 13.89 thousand per µL; Table S1, Supporting Information). In order to evaluate the effects produced by prolonged exposure of the blood to the stress induced by the magnetic catheter, further tests were performed by exposing the blood sample to 10 consecutive passages (considered as a “worst case” condition) within the retrieval catheter. Results confirmed no signs of hemolysis and platelet degradation: control and treated samples almost overlapped (differences can be ascribed to variability among samples; Table S2, Supporting Information).

The proposed intravascular magnetic tool showed a high particle capture efficiency (up to 94%) and the ability to keep this efficiency even upon multiple retrieval phases. These results demonstrated that the progressive accumulation of magnetic nanoparticles on magnets surface would not hamper the overall system efficiency, not affecting the ability to retrieve magnetic nano‐objects from the fluid stream. This paves the way for therapeutic protocols based on multiple infusions of high doses of magnetic therapeutic nanovectors.^32^, ^33^

In vitro validation revealed that the proposed solution is suitable for the devised application and that the developed FEM model represents a reliable tool to guide system design. The proposed approach could be also easily translated in the future to other body districts featured with terminal circulation, such as the pancreas. In this case, the need of more efficient targeted therapy strategies is even more demanding, due to the scarce efficacy of currently available surgical and pharmaceutical strategies in treating this cancer type. Proving the hemocompatibility and the overall safety of the proposed intravascular tool in vivo remains a challenge to address. Preliminary results on ex vivo whole blood samples revealed a small hemolysis, but further investigations are required to shed light also on possible platelet and leukocytes activation mechanisms.

However, some considerations can be made, supporting the potential of future in vivo validation and clinical adoption of the proposed magnetic retrieval strategy. In accordance with traditional chemotherapy cycles periodicity, consecutive catheterizations could be performed every 2–4 weeks. The minimal invasiveness of the proposed approach will make it suitable for such temporized therapeutic paradigm: in fact, the device will enter in contact with the vein endothelium only in correspondence to the insertion point and the anchoring balloon^34^; furthermore, the typical endothelium regeneration time is compatible with such time‐frame.^35^ The invasiveness of the proposed approach results minimal even if compared with transcatheter arterial chemoembolization (TACE), representing one of the most effective targeted chemotherapy approaches proposed until now, since no occlusion or embolization is needed. Retrieving therapeutics not accumulated within the tumor could also avoid liver failure and long‐term complications witnessed in the case of TACE due to the concentration of high doses of therapeutics.^36^

A system able to remove from the bloodstream the nanoagents not contributing to the therapy would enable to inject higher amounts of effective therapeutics, thus enhancing their accumulation at the tumor site without increasing undesired effects on healthy tissues. This approach paves the way to the exploitation of drug formulations (e.g., Cisplatinum and Doxorubicin) characterized by high tumor treatment efficacy, but unacceptably endangering healthy tissues.^37^, ^38^ Moreover, it can support related therapies such as magnetic hyperthermia, which success strongly depends on focused dose accumulation. Such a tool holds the potential to shift and influence also future steps and efforts in the field of nanomedicine: thanks to the ability to retrieve unused and unaccumulated nanovectors, the device allows to inject higher doses without running into severe side effects. This also makes nanovector circulation time (an important challenge on which current research efforts are focusing) a rather secondary aspect, being the accumulation targeted immediately after the injection, before retrieving. Finally, the proposed retrieval tool could enable the deployment of magnetic medical microrobots^16^, ^39^ to the clinical practice.^40^

## Experimental Section and Methods

*Magnetic Retrieval Modeling Methods*: Capturing low‐volume particles dragged under realistic blood flow conditions is an extremely challenging problem^17^ that has been addressed in different fields ranging from water purification^41^ to magnetic separation systems for biological applications.^42^ Usually, filter‐like structures and/or huge magnetic field generation apparatus are employed to produce magnetic field values sufficient to overcome the flow drag force. The employment of this kind of magnetic separation apparatus in an intracorporeal application is not straightforward due to dimensional constraints, biocompatibility issues, and need of compliance with the bloodstream. Due to size constraints and in order to avoid electromagnets heating, properly shaped and arranged miniature permanent magnets were employed.

A magnetic nanoparticle, dragged by a fluid and immersed in a magnetic field, travels with a speed resulting from two contributions: the first is produced by the fluid flow and the second is generated by the magnetophoretic force. Particle velocity *v*_p_ can be expressed as follows^17^:

(1) $$v_{p}\;=\;v\;+\;\zeta f\left(H\right)\left(H\cdot \nabla \right)H$$

where *v* is the fluid velocity and *H* is the applied magnetic field. ζ indicates the combination of drag and magnetic force acting on the particle and is expressed by Equation (2), whereas *f*(*H*), defined in Equation (3), consists of the contribution of nanoparticle magnetization and saturation due to the applied magnetic field.

(2) $$\zeta \;=\;\frac{\mu_{0}\left(1\;+\;\chi_{f}\right)}{6\pi \eta_{f}}\frac{V_{\mathrm{mag}}}{r_{h}}$$

(3) $$f\;\left(H\right)=\{\begin{array}{cc} \frac{3\left(\chi_{\text{p}}-\;\chi_{\text{f}}\right)}{\left(\chi_{\text{p}}-\;\chi_{\text{f}}\right)+3\left(1+\;\chi_{\text{f}}\right)} & \frac{M_{\text{sp}}}{H}\;>\;\frac{3\left(\chi_{\text{p}}-\;\chi_{\text{f}}\right)}{\left(\chi_{\text{p}}-\;\chi_{\text{f}}\right)+3\left(1+\;\chi_{\text{f}}\right)}\\ \frac{M_{\text{sp}}}{H} & \frac{M_{\text{sp}}}{H}\leq \;\frac{3\left(\chi_{\text{p}}-\;\chi_{\text{f}}\right)}{\left(\chi_{\text{p}}-\;\chi_{\text{f}}\right)+3\left(1+\;\chi_{\text{f}}\right)} \end{array}$$

In the previous equations, *µ_0_* is the magnetic vacuum permeability, χ_f_ and χ_p_ are the magnetic susceptibility of fluid and particles, respectively, η_f_ is the fluid dynamic viscosity, *V*_mag_ is the volume of the particle magnetic core, whereas *r*_h_ is the particle hydrodynamic radius. Lastly *M*_sp_ is the particle saturation magnetization.

The magnetophoretic component determines magnetic nanoparticles deviation toward the magnets and, as a consequence, nanoparticles capture. Preliminary numerical simulations were carried out by coding/integrating Equations (1)–(3) in Matlab (Matlab, MathWorks). Corresponding results showed that saturation occurs closer to the magnet edges, where the magnetophoretic action gets maximized by gradient effects (cf. Equation (1)).

The numerical approach was then extended to 2D axisymmetric domains (by exploiting the aforementioned commercial solver): the axisymmetric description permitted to contain computational costs while retaining relevant physical phenomena occurring in cylindrical vessels. In particular design parameters (magnet number, shape, size, and arrangement) were parametrically varied in order to assess the corresponding effect on capture efficiency.

Magnetic nanoparticles dragged by the bloodstream flowing in a rigid tube and eventually trapped by the embedded permanent magnets were modeled with the aim to quantify particle capture efficiency.

To depict this problem, three main physical effects were considered: 1) magnetic attraction exerted on the magnetic nanoparticles due to the permanent magnets. To model this effect, static magnetic fields produced by permanent magnets were calculated (magnetic fields – no Current Module in COMSOL Multyphysics); 2) fluidic drag force field due to blood flow in the magnetic module. According to the proposed intravascular device, the blood flowing in the vessel is canalized to pass across the retrieval magnetic module. The blood flow could be thus approximated to a laminar flow in a rigid channel (Laminar Flow Module in COMSOL Multyphysics); 3) particle trajectories due to the fluidic and magnetic force field, thus to drag and magnetophoretic force acting on them. Particle tracing enabled to quantify the number of particles retrieved from the bloodstream, thus the capture efficiency of the proposed device (Particle Tracing Module in COMSOL Multyphysics).

FEM simulations included two studies: a stationary study for magnetic fields and laminar flow analysis and a time‐dependent one, based on previous study solutions, for particle tracing. In this last implementation, the force field obtained by magnetic and fluidic modeling solutions were imported to define the drag force and magnetophoresis phenomena acting on the magnetic nanoparticles.

An extremely fine triangular mesh was used (10 µm maximum element size in the flow region, 50 µm maximum element size elsewhere). Independence studies were performed to setup the numerical solver parameters and obtain discretization‐independent results. The relevant adopted parameters are summarized in **Table** **1**.

**Table 1:** Main design and simulation parameters

Geometrical constraints	External diameter 3.6 mm	Internal channel 1 mm	Maximum length ≈40 mm
Blood modeling	Dynamic viscosity 35 cp	Density 1035 kg m^−3^	Average inlet speed 7 cm s^−1^
Permanent magnets	Residual magnetization 10^6^ A m^−1^	Minimum height 1 mm	Minimum wall thickness 0.6 mm
Magnetic nanoparticles	Diameter 10–500 nm	Relative magnetic permeability 10^3^	

The results obtained from the 2D axisymmetric simulations fully confirmed the preliminary numerical investigation: 1) capture enhanced closer to magnet edges; 2) nontrivial sizing of the gap because its narrowing implies an increased flow speed. Short permanent magnets (1 mm high) were thus chosen in order to minimize the axial encumbrance of the magnetic retrieval module.

*ICP‐MS Analysis*: In order to quantify the number of particles retrieved from the flow, the samples collected at the circuit output were analyzed through ICP‐MS. This technique relied on the combination among high‐temperature ICP source and an MS: the first converted the sample atoms into ions, whereas the latter detected these ions. The greatest advantage in using this technique lied in the possibility to detect and quantify metals in a sample with high accuracy and sensitivity even to very low concentrations. ICP‐MS was employed to quantify the amount of nanoparticles dispersed in the collected volume, thus not retrieved by the magnetic module. For each sample, the collected solutions were extensively sonicated and 5 µL was dissolved in 500 µL of hydrochloric acid for trace analysis. The solution was then digested in a microwave reactor (Discover SP‐D, CEM) for 20 min at 200 °C. After digestion, each sample was diluted with water to reach a 5 mL volume (ICP‐MS grade) and analyzed by ICP‐MS (Agilent Technologies 7700e Series ICP‐MS). All measurements were performed six times for each sample (Figure S4, Supporting Information). Iron content was determined by comparison with a standard curve. By considering a uniform distribution of nanoparticles in the collected volume, the amount of iron was derived and capture efficiency was calculated by comparing the amount of iron in the samples with the injected one.

Capture tests were carried out on four magnetic module prototypes for each particle dimension and four consecutive tests were performed on each prototype. For each collected sample, six 5 µL quotes were analyzed through ICP‐MS and statistical data analysis enabled to obtain the presented results.

## Conflict of Interest

The authors declare no conflict of interest.

## Supporting information

Supplementary

Supplementary

Supplementary

Supplementary

Supplementary
