# Supplementary material for: An Intravascular Magnetic Catheter Enables the Retrieval of Nanoagents from the Bloodstream
Source: Adv Sci (Weinh). 2018 Aug 1;5(9):1800807. doi: 10.1002/advs.201800807 (PMC6145422; doi:10.1002/advs.201800807)
Supplement: Supplementary file 1 — Supplementary [file ADVS-5-1800807-s001.pdf]

Copyright WILEY-VCH Verlag GmbH & Co. KGaA, 69469 Weinheim, Germany, 2018.

Supporting Information

for *Adv. Sci.,* DOI: 10.1002/advs.201800807

An Intravascular Magnetic Catheter Enables the Retrieval of Nanoagents from the Bloodstream

Veronica Iacovacci, Leonardo Ricotti, Edoardo Sinibaldi,* Giovanni Signore, Fabio Vistoli, and Arianna Menciassi*

Supporting Information

An intravascular magnetic catheter enables the retrieval of nanoagents from the bloodstream

Veronica Iacovacci*, Leonardo Ricotti, Edoardo Sinibaldi*, Giovanni Signore, Fabio Vistoli, and Arianna Menciassi

SUPPLEMENTARY FIGURES

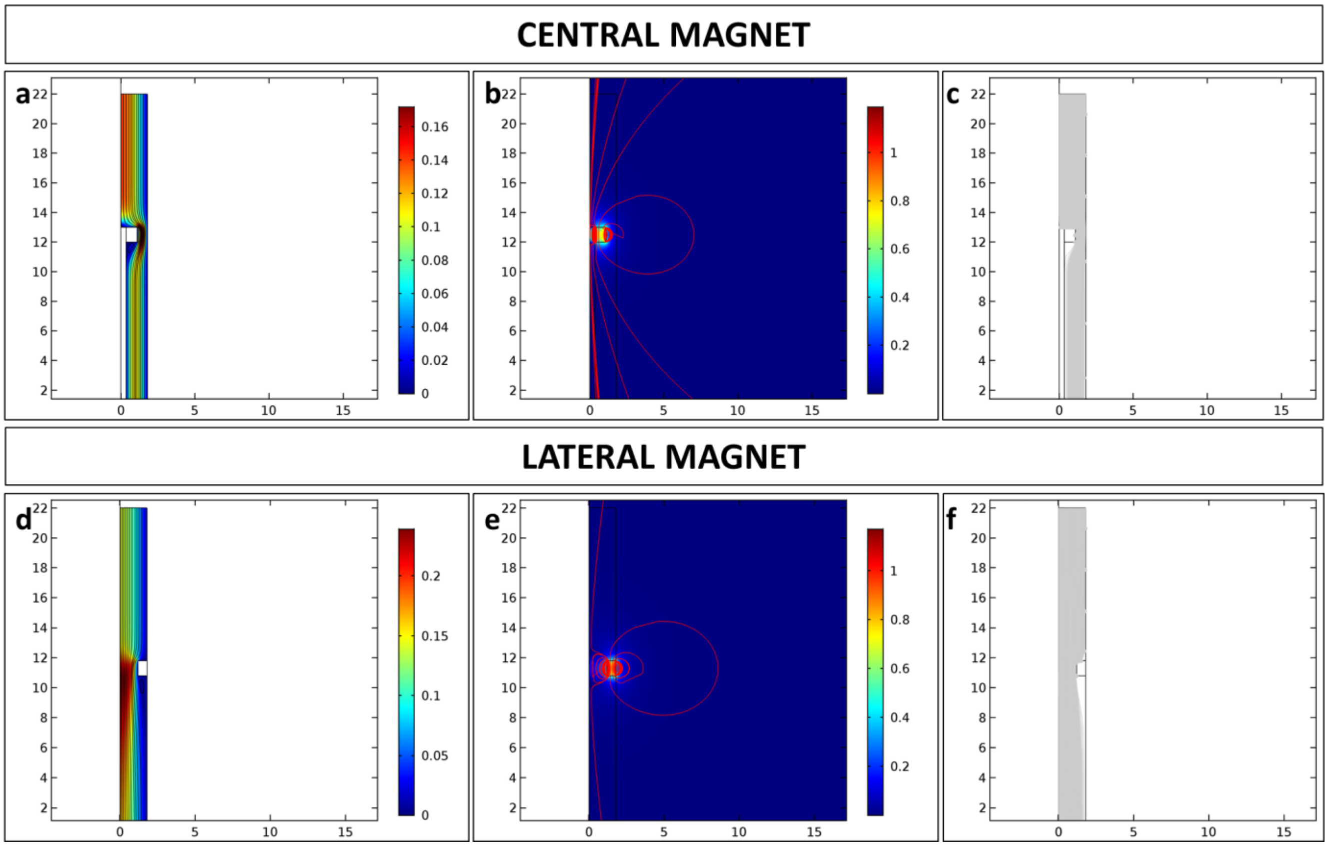

Figure S1 Multiphysics simulation results when considering a single ring-shaped permanent magnet placed either at the center or along the side wall of the magnetic module. For each configuration fluidic field (a,d), magnetic field with streamlines (b,e) and particle trajectories (c,f) are reported. For tracing particle trajectories, 100 particles were considered.

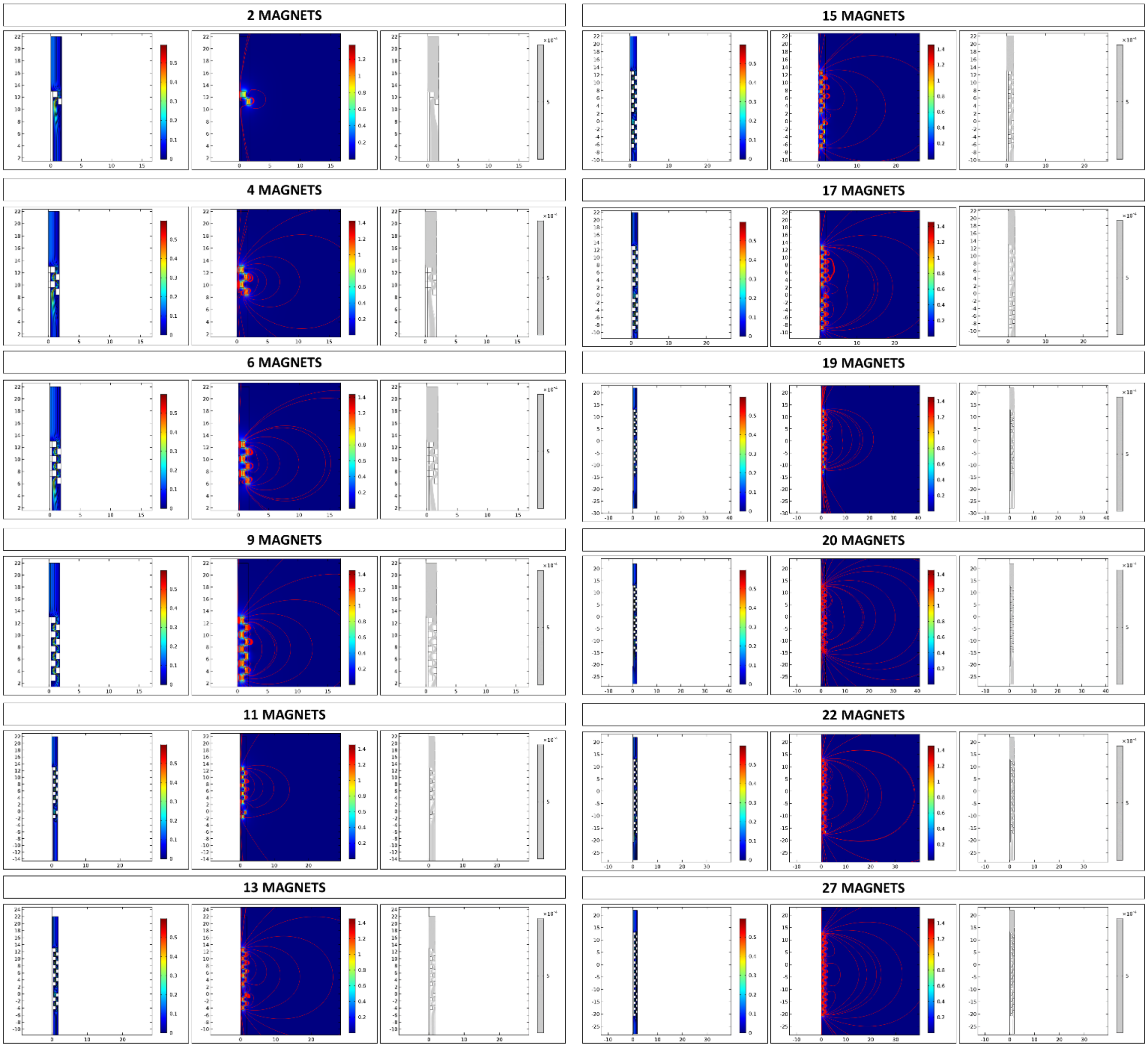

Figure S2 Multiphysics simulation results when varying the number of the embedded permanent magnets from 2 to 27. For all configurations, fluidic field, magnetic field (with streamlines) and particles trajectories are reported. For tracing particle trajectories, 100 particles were considered.

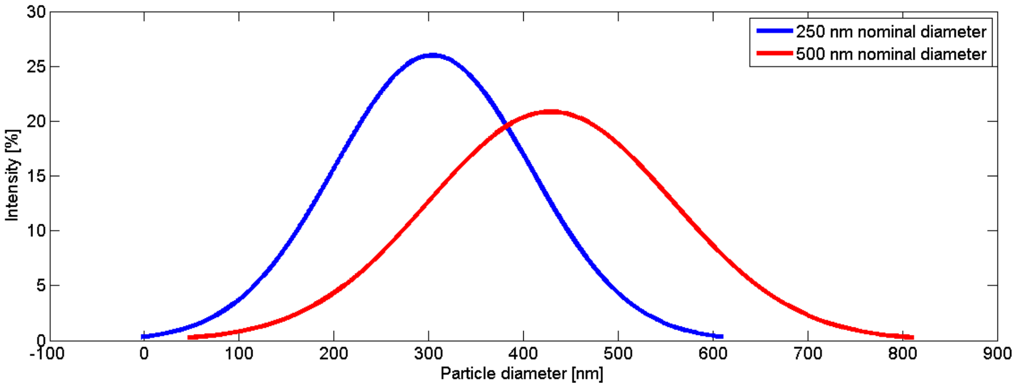

Figure S3 Dynamic light scattering results for the two classes of magnetic nanoparticles. Recorded signal intensity features a distribution close to a Gauss plot with a certain deviation with respect to the peak value.

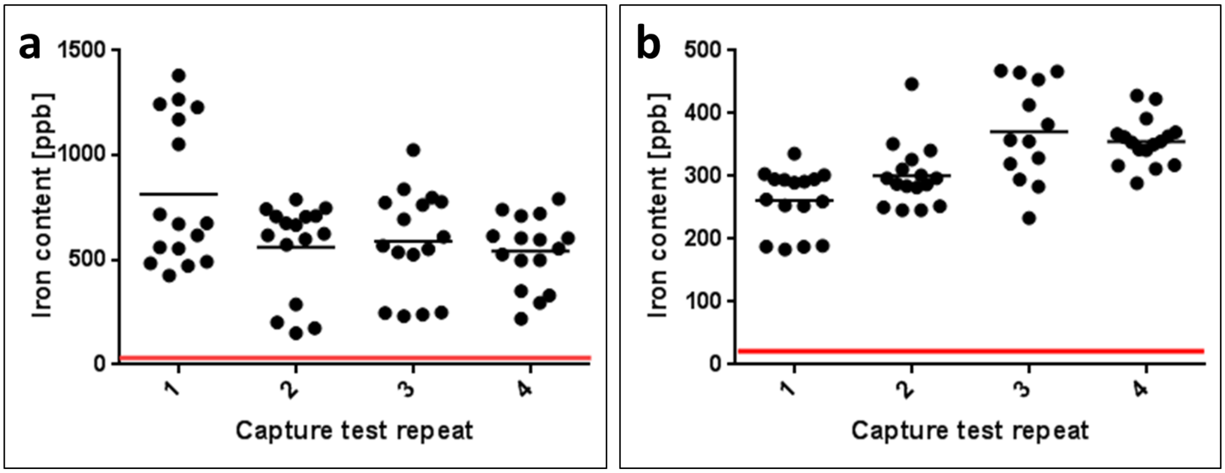

Figure S4 ICP-MS analysis results for 250 (a) and 500 (b) nm nominal diameter nanoparticles. Iron content in 5 µL samples from the collected solutions is plotted versus the capture test repetition. Results are expressed in terms of counts per billion and the red line represents the basal value corresponding to Iron content in the blood-mimicking solution.

	CONTROL				SINGLE PASSAGE			
	TEST 1	TEST 2	TEST 3	MEAN	TEST 1	TEST 2	TEST 3	MEAN
RBC [10^6/µL]	7.4	7.45	7.42	7.42±0.02	6.99	7.35	7.35	7.23±0.21
Hct [%]	43.1	43.1	43	43.07±0.06	41.9	43.1	42.8	42.6±0.62
Plt [10^3/µL]	380	355	385	373.33±16.07	346	371	369	372±13.89

Table S 1 Ex vivo blood sample exams results: comparison among control (samples just passed across the commercial catheter, without any modification) and single passage samples (across the retrieval catheter, i.e. that one modified by adding the magnetic module).

	CONTROL	10 PASSAGES			
		TEST 1	TEST 2	TEST 3	MEAN
RBC [10^6/μL]	8.10	8.45	8.2	7.95	8.2±0.25
Hct [%]	47	49.4	48	47.1	48.17±1.16
Plt [10^3/μL]	296	328	336	339	334.33±5.69

Table S 2 Ex vivo blood sample exams results: comparison among control (samples just passed across the commercial catheter) and ten-passages samples (10 times across the retrieval catheter).
